# Translatability of WGS typing results can simplify data exchange for surveillance and control of *Listeria monocytogenes*

**DOI:** 10.1099/mgen.0.000491

**Published:** 2020-12-04

**Authors:** Stefanie Lüth, Carlus Deneke, Sylvia Kleta, Sascha Al Dahouk

**Affiliations:** ^1^​ National Reference Laboratory for *Listeria monocytogenes*, German Federal Institute for Risk Assessment, Department of Biological Safety, Berlin, Germany; ^2^​ Institute of Biology, Freie Universität Berlin, Department of Biology, Chemistry and Pharmacy, Berlin, Germany; ^3^​ Study Centre for Genome Sequencing and Analysis, German Federal Institute for Risk Assessment, Department of Biological Safety, Berlin, Germany; ^4^​ RWTH Aachen University Hospital, Department of Internal Medicine, Aachen, Germany

**Keywords:** core genome MLST, genomic epidemiology, outbreak, single nucleotide polymorphism, standardisation, whole genome sequencing

## Abstract

Where classical epidemiology has proven to be inadequate for surveillance and control of foodborne pathogens, molecular epidemiology, using genomic typing methods, can add value. However, the analysis of whole genome sequencing (WGS) data varies widely and is not yet fully harmonised. We used genomic data on 494 *Listeria monocytogenes* isolates from ready-to-eat food products and food processing environments deposited in the strain collection of the German National Reference Laboratory to compare various procedures for WGS data analysis and to evaluate compatibility of results. Two different core genome multilocus sequence typing (cgMLST) schemes, different reference genomes in single nucleotide polymorphism (SNP) analysis and commercial as well as open-source software were compared. Correlation of allele distances from the different cgMLST approaches was high, ranging from 0.97 to 1, and unified thresholds yielded higher clustering concordance than scheme-specific thresholds. The number of detected SNP differences could be increased up to a factor of 3.9 using a specific reference genome compared with a general one. Additionally, specific reference genomes improved comparability of SNP analysis results obtained using different software tools. The use of a closed or a draft specific reference genome did not make a difference. The harmonisation of WGS data analysis will finally guarantee seamless data exchange, but, in the meantime, knowledge on threshold values that lead to comparable clustering of isolates by different methods may improve communication between laboratories. We therefore established a translation code between commonly applied cgMLST and SNP methods based on optimised clustering concordances. This code can work as a first filter to identify WGS-based typing matches resulting from different methods, which opens up a new perspective for data exchange and thereby accelerates time-critical analyses, such as in outbreak investigations.

## Data Summary

The authors confirm all supporting data, codes and protocols have been provided within the article or through supplementary data files.

Sequencing data have been deposited in the European Nucleotide Archive (ENA) at EMBL–EBI under the accession number PRJEB38495, except for isolate 16-LI00360-0, which is available under the accession number ERS4418852 (SAMEA6659390).

**Impact Statement**

For effective surveillance and control of human listeriosis, not only comprehensive molecular typing of *Listeria monocytogenes* isolates from food, food processing environments and clinical cases, but also communication of results between different sectors (food safety, public health) and countries is needed. The currently available procedures for WGS-based typing are diverse and not yet fully harmonised. The ideal way to go for the future will be the harmonisation of methods between different laboratories to enable seamless data exchange. However, until a generally accepted solution has been found, an interim solution has to be established. We therefore compared the results of the most commonly used genotyping tools for *L. monocytogenes *, and developed a translation code for the identification of typing matches resulting from different methods. This approach opens up a new perspective for the exchange of WGS analysis results.

## Introduction

*Listeria monocytogenes* is the causative agent of the infectious disease listeriosis. While infections may be asymptomatic in otherwise healthy individuals, vulnerable population groups, like immunocompromised or elderly people, pregnant women and newborns, are likely to suffer from severe clinical symptoms, sometimes with a fatal outcome [1]. Although listeriosis is comparatively rare, a hospitalisation rate of 98.6 % and a case fatality rate of 13.8 % in the European Union (EU) in 2017 clearly show the serious public health hazards [2]. The vast majority of *L. monocytogenes* infections are foodborne [3]. Consequently, tracing back clinical cases to contaminated food products is one of the key requirements for disease control. However, classical epidemiology alone has proven to be inappropriate for that purpose. The main reasons for this are the very broad range of potentially affected food vehicles and the long incubation period and severity of disease, which complicate patient interviews on food consumption [4–7]. As a result, molecular typing methods have long been applied in *L. monocytogenes* surveillance and outbreak investigations. During recent years, whole genome sequencing (WGS) has revolutionised this field through its unprecedented resolution [8–11].

There are basically two different approaches for WGS-based typing. The first approach is a gene-by-gene comparison where the analysis focuses on allele differences. An example of this approach is core genome multilocus sequence typing (cgMLST), an extension of classical MLST to a larger set of genes that is shared among members of a single species. In the case of *L. monocytogenes *, two main cgMLST schemes are currently in use. One comprises 1701 loci and is built into the software Ridom SeqSphere+ [12], whereas the other one comprises 1748 loci and is built into the software BioNumerics [13]. This incorporation of cgMLST schemes into commercial tools with a graphic user interface has the great advantage of a straightforward operation, including for users lacking bioinformatics skills. However, cost-intensive software licenses might not be affordable for all users. In these cases, it is helpful that both cgMLST schemes are also publicly available and can be used within open-source tools such as the Blast-score-ratio-Based Allele Calling Algorithm (chewBBACA [14]). Although a little bioinformatics training is required, tools like this provide a low-cost alternative. The second approach for WGS-based typing is the single nucleotide polymorphism (SNP) analysis. In this case, single nucleotide variations are used as a distance measure between bacterial sequences. Both, commercial (e.g. BioNumerics) and open-source (e.g. Snippy [15]) solutions are available for analysis. SNP analysis is commonly based on a comparison against a selected reference genome. However, the genome chosen as reference can affect analysis results [16, 17].

For effective surveillance and control of human listeriosis, not only comprehensive molecular typing of *L. monocytogenes* isolates from food, food processing environments and clinical cases, but also communication of results between different sectors (food safety, public health) and countries is needed. However, procedures for WGS-based typing are diverse and not yet fully harmonised. Starting from the sequencing protocol through quality filtering (e.g. read trimming) to algorithms for assembly, mapping or variant calling and finally distance assessment, there is considerable space for variation. The ideal way to go for the future will be the harmonisation of all these methods between different laboratories to enable the seamless exchange of analysis results. To date, several international initiatives have been commenced to deal with this issue [18–20]. However, until a generally accepted solution has been found, an interim solution is urgently needed.

In order to assess the transferability of results, we compared the most commonly used WGS-based typing methods for *L. monocytogenes *. Our aim was to provide a translation code as a first filter for the identification of typing matches resulting from the different methods. In addition, we describe a procedure that can also be applied to the comparison of other methods.

## Methods

### Study dataset

A total of 494 isolates from ready-to-eat food products and food processing environments sampled in official controls in 2016 were selected from the strain collection of the German National Reference Laboratory for *L. monocytogenes* as a representative dataset for the population structure of *L. monocytogenes* in the food chain in Germany.

### Bacterial strain cultivation

Pure cultures of *L. monocytogenes* isolates were routinely stored at −80 °C in brain heart infusion medium with 20 v/v % glycerol. Prior to downstream analysis, bacteria were plated onto Sheep Blood Agar and incubated overnight at 37 °C.

### Genomic DNA extraction and whole genome sequencing (WGS)

Overnight cultures of *L. monocytogenes* strains were harvested and lysed following the PulseNet standardised laboratory protocol for WGS of Gram-positive bacteria (https://www.cdc.gov/pulsenet/pdf/pnl32-miseq-nextera-xt.pdf). For DNA extraction, the QIAamp DNA Mini Kit (Qiagen) was used following the manufacturer’s instructions. Purity of extracted DNA (OD_260:280_ and OD_260:230_ ratio) was measured with the NanoDrop spectrophotometer (Thermo Fisher Scientific) and extracted DNA was quantified using the Qubit dsDNA BR Assay Kit with a Qubit 2.0 fluorometer (Invitrogen). Sequencing libraries were constructed with the Nextera XT Sample Preparation Kit (Illumina) for sequencing in paired-end mode with 2×300 bp reads on an Illumina MiSeq sequencer using the MiSeq Reagent v3 600-cycle Kit (Illumina).

### Sequencing quality control and genome assembly

Raw sequencing reads were quality checked using FastQC version (v) 0.11.5 [21] and trimmed using Trimmomatic v 0.36 [22]. Subsequently, trimmed reads were assembled and analysed using the pipeline Assembly-based QUality Assessment for Microbial Isolate Sequencing (AQUAMIS) v 0.9.0 at default parameters [23]. This pipeline includes the tools unicycler v 0.4.4 for assembly and assembly polishing, mash v 2.1 for reference search, and quast v 4.6.3 for assembly quality control. Genome assemblies obtained from the AQUAMIS pipeline served as a starting point for cgMLST-based typing, whereas trimmed reads were used as the basis for SNP analysis. Detailed information on tools and parameters can be found in Supplementary File S1 (available in the online version of this article).

### Classical multilocus sequence typing (MLST)

Classical seven-gene MLST sequence types (STs) and corresponding MLST clonal complexes (CCs) were determined from assembled draft genomes according to the scheme available at https://bigsdb.pasteur.fr/listeria/listeria.html using Ridom SeqSphere+ (Ridom).

Closed genomes of *L. monocytogenes* available at NCBI were analysed with the software mlst [24] and a reference genome was chosen for each MLST CC (https://github.com/crarlus/refseq-MLST/).

### Core genome (cg) MLST analysis

#### Ridom SeqSphere+

Assembled draft genomes were analysed in Ridom SeqSphere+with the ‘Process assembled genome data’ function at default parameters for *L. monocytogenes *. The integrated 1701 loci scheme was used [12]. A cgMLST allele coverage of at least 98 % was set as quality threshold, assuming that this value is representative of the entire genome quality [25]. If the threshold was not reached, sequencing was repeated. Resulting allele profiles were exported in tsv format. This method will be referred to as Ridom_Ruppitsch.

#### BioNumerics

The WGS tools plugin of BioNumerics v 7.6.3 (Applied Maths) was used for analysis with the integrated 1748 loci cgMLST scheme [13]. Resulting cgMLST allele profiles were exported in tsv format. This method will be referred to as BioNumerics_Moura.

#### chewBBACA

chewBBACA is a freely available software suite that allows scheme creation, allele calling and scheme evaluation [14]. Allele calling starts with the identification of coding sequences (CDS) using prodigal [26]. If an exact match to the allele database is found, the corresponding allele number is assigned. Otherwise, a blastp score ratio (BSR) approach evaluates whether a novel allele is present, or no allele can be inferred. Newly inferred alleles are updated in a local allele database.

Here, we used the pipeline chewieSnake [27] that calls alleles for a set of samples using chewBBACA v 2.0.12, combines their allele profiles and infers an allele distance matrix as well as a minimum spanning tree using GrapeTree v 1.4.1 [28]. Subsequently, samples can be hierarchically clustered and a cgMLST report is compiled. As we used chewieSnake with the cgMLST scheme developed by Ruppitsch and colleagues [12], this method will be referred to as chewBBACA_Ruppitsch.

### SNP analysis

#### Reference genomes

The application of three different kinds of reference genomes was compared: 1) general, species-specific, closed; 2) subgroup-specific, closed; 3) subgroup-specific, draft. The genome of the *L. monocytogenes* reference strain EGDe (NC_003210.1) was used as general reference. Isolates were assigned to subgroups according to MLST CCs. As far as possible, MLST CC-specific closed reference genomes were selected as described by the European Food Safety Authority [16]. To identify reference genomes for those MLST CCs for which the European Food Safety Authority had not specified one, closed genomes of *L. monocytogenes* available at NCBI were subjected to MLST CC determination. In the case of more than one closed genome per MLST CC, the reference genome was selected randomly. For MLST CC-specific draft reference genomes, draft genomes with the best assembly quality (highest N50, lowest number of contigs) per MLST CC were selected from our dataset. Only MLST CCs containing more than five isolates and with a closed reference genome available in NCBI were included in MLST CC-specific analyses.

#### BioNumerics

For SNP analysis in BioNumerics, the basic version of BioNumerics v 7.6.3 without the WGS tools plugin was used. Strict filtering of SNPs at software default settings was applied. This method will be referred to as SNP_BioNumerics.

#### Snippy

Snippy was chosen as a representative open-source SNP pipeline since it is recognised as one of the most reliable SNP pipelines [29].

SNPs were detected with the variant calling pipeline snippy-snake [30]. In short, SNPs were called with snippy v 4.0 [15], the core alignment was determined using snippy-core and the SNP distance matrix using snp-dists [31]. Subsequently, the pipeline clustered all samples into cluster types for a range of thresholds using hierarchical clustering and generated a SNP report. This method will be referred to as SNP_Snippy.

#### Filtering of isolates for MLST CC-specific analyses

While generally applicable typing methods may give an adequate overview, it can be useful to repeat certain analyses only for a subgroup of isolates to gain deeper insights. We used MLST CC-specific analysis for this purpose. However, isolates belonging to different MLST CCs may vary in diversity, for example depending on the number of individual STs within the CC. This can result in exceptionally large SNP distances, which will distort the results. Actually, the establishment of the largest core genome is required for detailed SNP analysis. Therefore, after initial SNP analysis, very distantly related isolates within each MLST CC were identified (>800 SNPs in SNP_BioNumerics and >18 000 SNPs in SNP_Snippy) and excluded from further MLST CC-specific analyses.

### Properties and correlations of distance matrices

For all selected cgMLST methods, distance matrices were calculated with GrapeTree v 1.4.1 [28] (using the option ‘--missing 0’ to deal with missing loci) based on allele profiles. Distance matrices for SNP analyses were used as yielded from primary analysis.

All downstream analyses from distance matrices were performed in R using the packages *plyr*, *reshape2* and *ggplot2*. Distance matrices were linearized and sorted to compare pairwise distances. For MLST CC-specific analyses, the resulting sub-settings of distance matrices per MLST CC were merged to one. Boxplots were generated to visualise the magnitude of detected distances and Spearman correlation was used to quantify the similarity of pairwise distances between different methods. Correlations were visualised using the package *corrplot*.

### Concordance of clustering

Distance matrices were used for single linkage clustering at different threshold values in R. The clustering results dependent on methods and thresholds served as inputs for the Comparing Partitions online tool available at http://www.comparingpartitions.info [32]. The adjusted Wallace coefficient was selected as pairwise agreement measure because it directly indicates the concordance between clusters. The coefficient can be regarded as the probability that a cluster calculated by method 1 matched that calculated by method 2, and vice versa. Always two adjusted Wallace coefficients deriving from two comparison directions were determined.

#### Establishment of a translation code between methods

We assessed the degree of concordance between three different cgMLST methods (comprising three software solutions and two cgMLST schemes) and six different SNP methods (two software solutions with three types of reference genomes each). Our aim was to define threshold values that can be communicated for a comparable interpretation of clustering results. As they are epidemiologically well defined, we chose two published allele distance thresholds for the cgMLST schemes (seven and ten allele differences, referred to in previous work [12, 13]) as references to establish our translation code. More precisely, the clustering information derived from one of the three cgMLST methods with one of the two threshold values was set as a reference and was compared with the clustering at various threshold values in a comparison method (other cgMLST method or SNP method) using the Comparing Partitions online tool as described above. The threshold value of the comparison method, at which the sum of the two adjusted Wallace coefficients reached a maximum, was defined as the ‘adjusted threshold’.

#### Practical test of the translation code

The translation code was tested with the cgMLST dataset retrieved from BioNumerics_Moura at an allele threshold of seven as reference method. The five largest clusters, for which also MLST CC-specific analyses had been performed, were selected. Thus, one cgMLST cluster each from MLST CC9, CC121, CC3, CC8 and CC2 was included in our analysis. One isolate per cgMLST cluster was randomly selected and used for cluster search at adjusted threshold values in the other methods. This approach reflects the generally valid workflows during international disease outbreaks, when the sequence of an individual reference isolate is shared between laboratories as the basis for local cluster identification.

## Results

### Quality control

All 494 isolates were sequenced with coverage between 32 and 231 (median 99). Raw reads could be assembled into 15 to 72 contigs (median 29) with an N50 between 9.6×10^4^ and 1.5×10^6^ (median 3.6×10^5^). Median cgMLST allele coverage using Ridom SeqSphere+was 99.8 %.

### Comparison based on distance matrices

#### Generally applicable methods

**Fig. 1.**
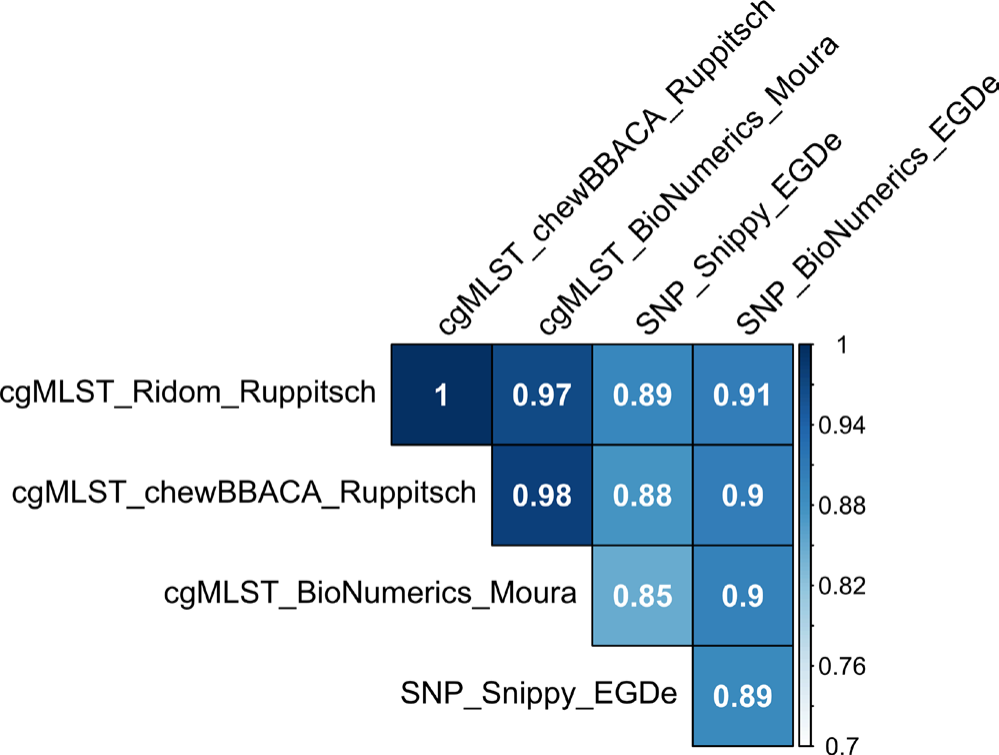
Correlations of generally applicable typing methods, based on linearized distance matrices. Colour scale indicates the strength of correlation.

In cgMLST analysis, pairwise allele distance between isolates ranged from 0 to 1687 (median 1347) using Ridom_Ruppitsch, from 0 to 1687 (median 1351) using chewBBACA_Ruppitsch and from 0 to 1740 (median 1409) using BioNumerics_Moura. The differences in pairwise distances resulting from Ridom_Ruppitsch and BioNumerics_Moura varied between −89 and 24 (median −55), from chewBBACA_Ruppitsch and BioNumerics_Moura between −87 and 27 (median −54), and from Ridom_Ruppitsch and chewBBACA_Ruppitsch between −12 and 4 (median −1). Method correlations were 0.97 and 0.98 using different cgMLST schemes and 1 with the same scheme (Fig. 1). A visual comparison between distances derived from different methods can be found in Supplementary file 2.

Pairwise SNP distance between isolates with EGDe as the reference genome ranged from 0 to 12 694 (median 3504) using SNP_BioNumerics and from 0 to 107 646 (median 26 884) using SNP_Snippy. Method correlation was 0.89.

Correlations of cgMLST_Ridom_Ruppitsch and cgMLST_chewBACCA_Ruppitsch were 0.89 and 0.88 to SNP_Snippy_EGDe and 0.91 and 0.9 to SNP_BioNumerics_EGDe, respectively (Fig. 1). Correlation of cgMLST_BioNumerics_Moura was 0.85 to SNP_Snippy_EGDe and 0.9 to SNP_BioNumerics_EGDe.

#### Subgroup (MLST CC)-specific methods

The 494 isolates belonged to 39 different MLST CCs (Tables S1 and S2), out of which 19 MLST CCs contained at least five isolates, but a closed reference genome was only available for 16 of them at NCBI. Accordingly, 409 isolates were selected for initial MLST CC-specific analyses. After filtering out those isolates with too large SNP distances within an MLST CC, 394 isolates from 15 different MLST CCs were left (Table 1). Filtered isolates came from CC8 (*n*=3), CC4 (*n*=1) and CC14 (*n*=9). As for CC14, only two isolates were left after filtering, the entire MLST CC was excluded from further analyses.

**Table 1.** MLST CCs and references used for MLST CC-specific analyses (sorted by frequency in our dataset)

MLST CC	Closed reference (GenBank Accession)	Draft reference	Coverage	Contigs
CC121	HG813249	16-LI01132-0	91	21
CC9	FR733649	16-LI00873-0	77	17
CC8	CP006862	16-LI00415-0	84	19
CC2	CP006046	16-LI01038-0	119	25
CC3	CP006594	16-LI00227-0	148	27
CC1	AE017262	16-LI00258-0	61	19
CC37	CP011397	16-LI00295-0	113	20
CC6	CP006047	16-LI00782-0	85	16
CC5	CP006592	16-LI00750-0	133	21
CC101	CP025221	16-LI00284-0	117	20
CC18	CP020830	16-LI00319-0	119	15
CC155	CP002004	16-LI00862-0	90	25
CC224	CP016629	16-LI00391-0	91	24
CC7	CP002002	17-LI00007-0	112	21
CC4	FM242711	16-LI00480-0	93	27

**Fig. 2.**
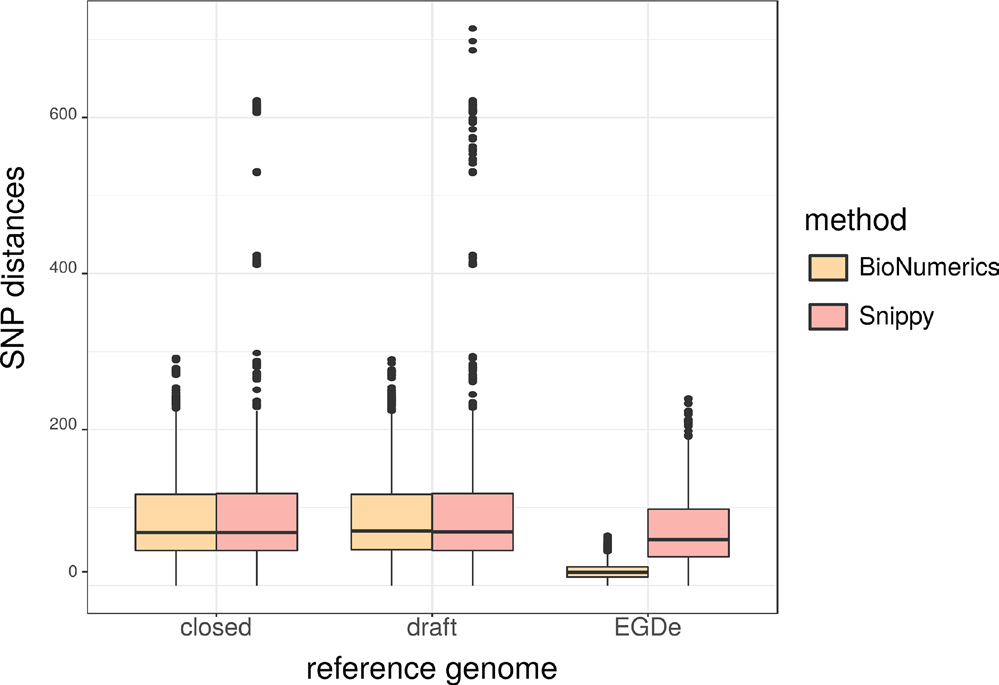
Boxplot of SNP distances from BioNumerics and Snippy using different reference genomes for SNP analysis (applied to a subset of 394 isolates of 15 different MLST CCs), based on linearized distance matrices.

In SNP_BioNumerics, use of an MLST CC-specific closed reference genome led to pairwise SNP distances between 0 and 292 (median 68), whereas use of a specific draft reference genome yielded 0 to 290 (median 70) pairwise SNP distances (Fig. 2). Applying SNP_Snippy, SNP distances with a specific closed reference genome ranged between 0 and 622 (median 68) and between 0 and 714 (median 69) with a specific draft reference genome. In the MLST CC-specific analyses with EGDe as a reference, SNP distances were 0 to 64 (median 17) using SNP_BioNumerics and 0 to 240 (median 59) using SNP_Snippy.

The median ratio of pairwise distances resulting from SNP analysis with the same software but with a closed or draft specific reference genome was 1. The median ratio between a closed or a draft specific reference and EGDe was 3.8 and 3.9, respectively, using SNP_BioNumerics, and 1.2 using SNP_Snippy. When EGDe was used as reference genome, the median ratio of pairwise distances between SNP_Snippy and SNP_BioNumerics was 3.1.

**Fig. 3.**
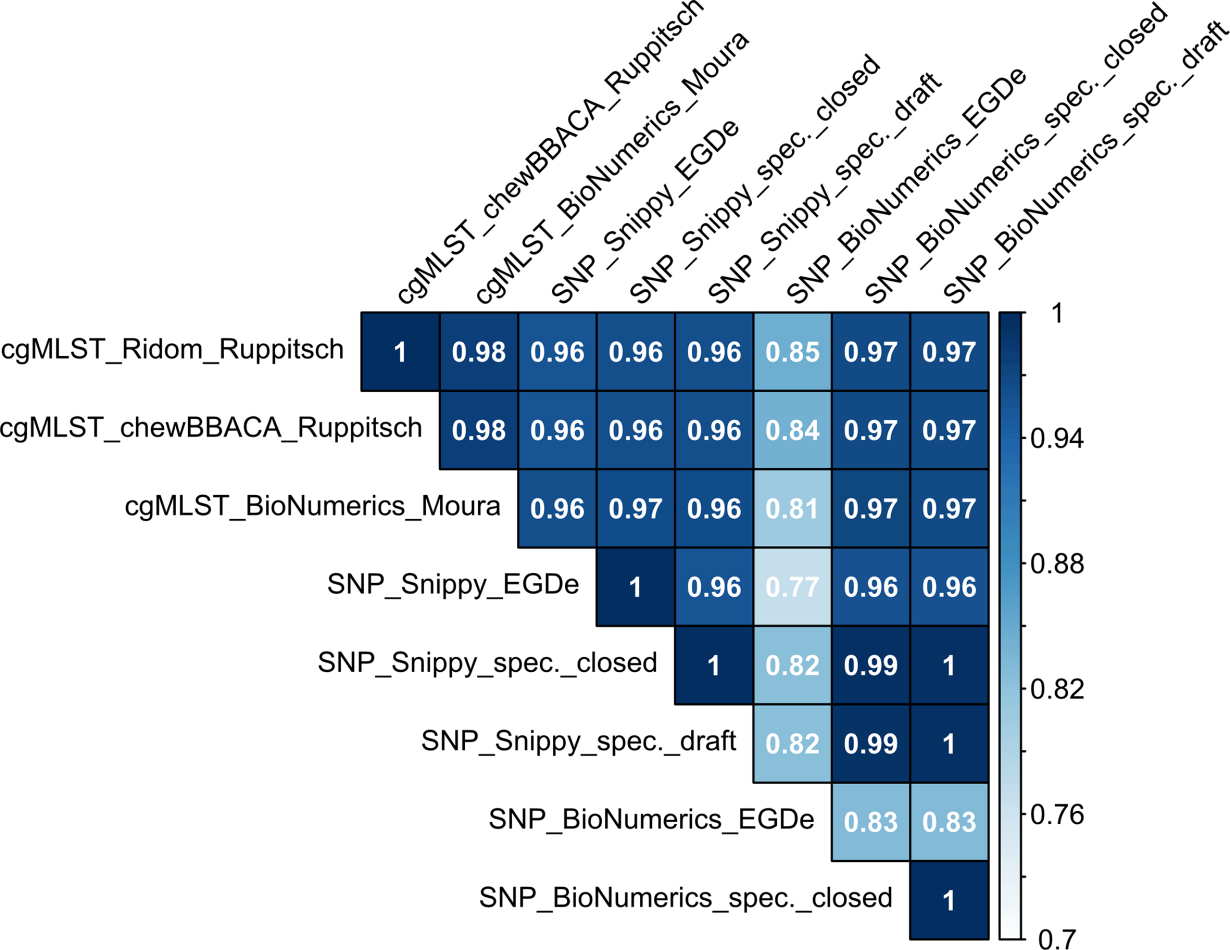
Correlations of MLST CC-specific typing methods (applied to a subset of 394 isolates of 15 different MLST CCs), based on linearized distance matrices. Colour scale indicates the strength of correlation.

Overall, there was near perfect (0.99) to perfect (1) correlation between different software and closed or draft specific reference genome usage in SNP analysis. Lowest method correlations were found with SNP_BioNumerics when using EGDe as reference (0.77 to 0.85). All other correlations were larger than 0.96 (Fig. 3).

#### Distances to the reference and size of the core genome

When having a more detailed look into the results from SNP_Snippy (Table S3), isolates had a smaller SNP distance to the draft than to the closed MLST CC-specific reference genomes. On the one hand, more SNP positions were missing when the isolate reads were mapped to the closed references. On the other hand, however, the size of the closed reference genomes tended to be larger than that of the draft ones (by 26 kbp on average). Altogether, the core genome size (defined as the number of positions in the reference that are neither missing nor masked in any of the isolate’s mapping to the reference) was in the end very similar between draft (median size 2 809 303 bp) and closed reference (median size 2 802 508 bp) genomes. In SNP_Snippy, the isolates had a distance of 25 000 SNPs to the EGDe reference on average. Furthermore, we observed a substantial increase in the number of missing as well as masked positions compared with the specific reference genomes. Therefore, the core genome size when using EGDe as reference was only 2 281 008 bp.

### Comparison of clustering

#### cgMLST methods

**Fig. 4.**
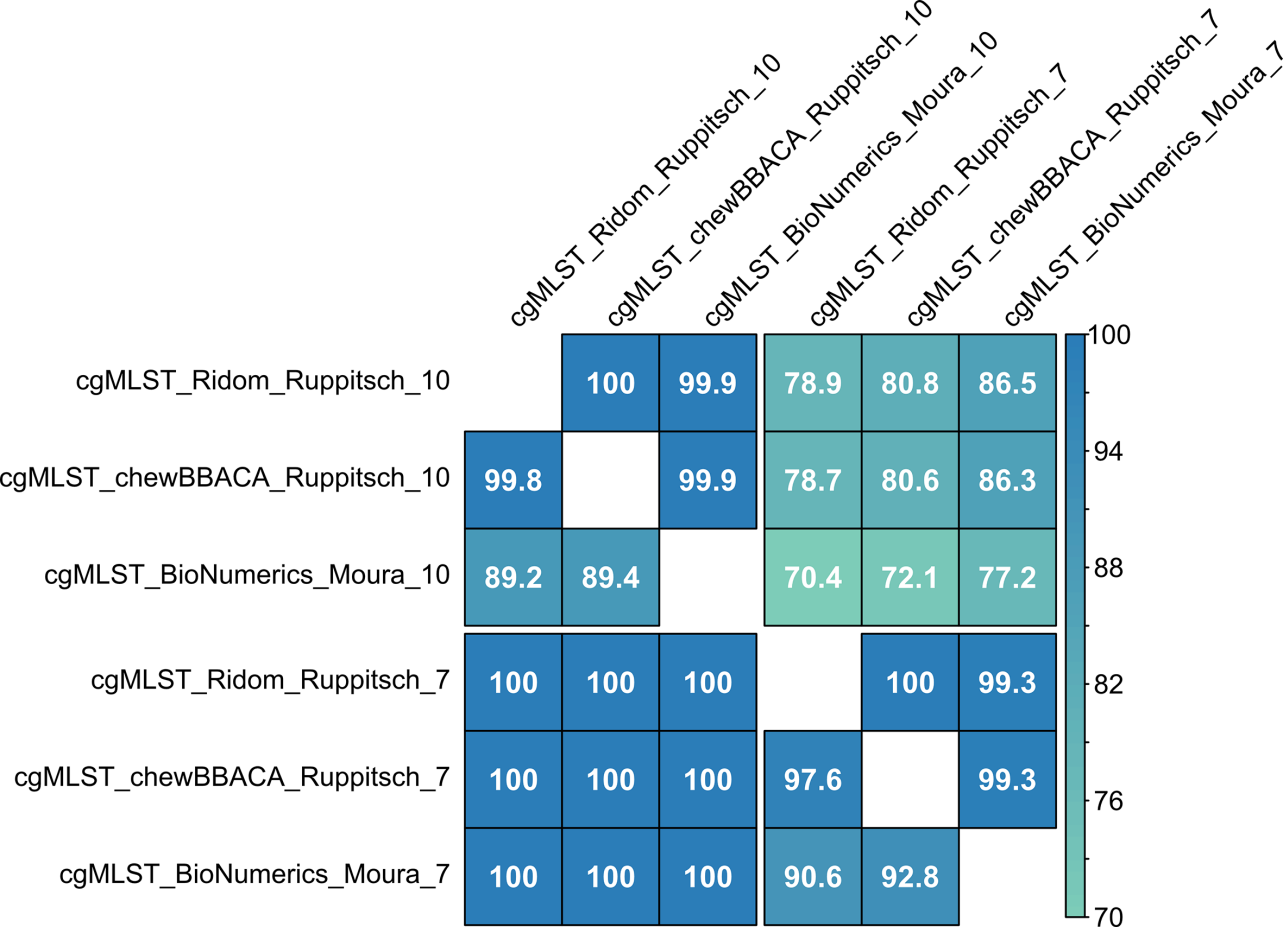
Matrix of adjusted Wallace coefficients (direction-dependent values) for cgMLST methods at common thresholds (seven and ten alleles). Colour scale indicates percentage of concordance.

To compare the clustering of isolates, threshold values published for the two cgMLST schemes were applied to the different cgMLST approaches. For the Ruppitsch scheme this is ten alleles [12], and for the Moura scheme seven alleles [13] between neighbouring isolates. Agreement was perfect when comparing clusters at a seven-allele threshold with clusters at a ten-allele threshold (adjusted Wallace coefficient 100 %). The other way around (from ten to seven), however, concordance was only between 70.4 and 86.5 % (Fig. 4). When using the same threshold values for different methods, overall concordance was higher than with different thresholds. At a threshold of seven alleles, concordance was 97.6 and 100 % when using the same cgMLST scheme in different software and between 90.6 and 92.8 and 99.3 % (depending on the direction) for different schemes. At a threshold of ten alleles, concordance was 99.8 and 100 % when using the same cgMLST scheme in different software and between 89.2 and 89.4 and 99.9 % (depending on the direction) for different schemes.

#### Optimisation of clustering and translation code between cgMLST and SNP methods

**Fig. 5.**
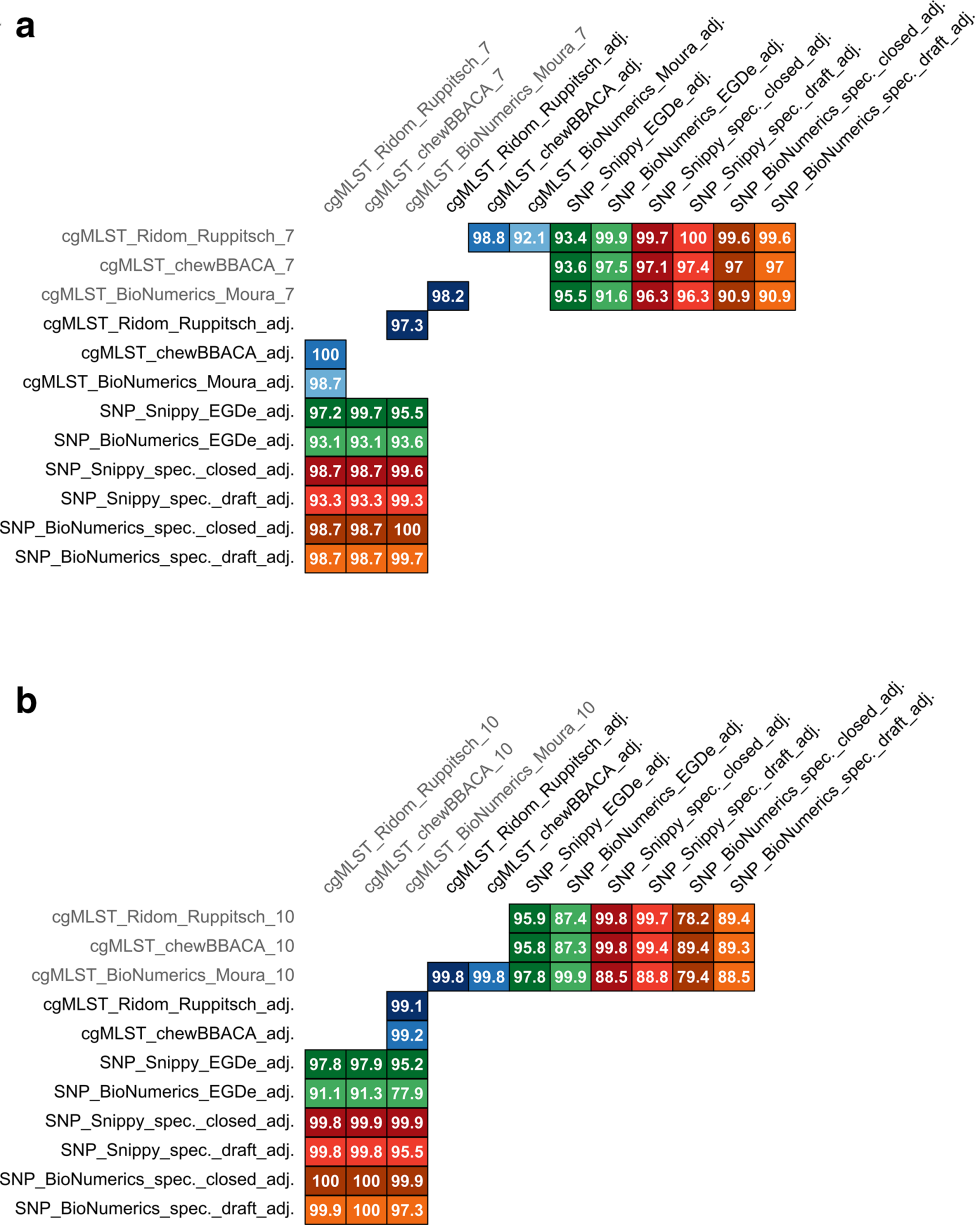
Adjusted Wallace coefficients (direction-dependent values) at optimised clustering thresholds. (a) Threshold seven alleles, (b) Threshold ten alleles. Grey text colour indicates that the method was used as the reference for threshold adjustment. Percentage values of concordance are presented. Each method has a specific colour and rows and columns of the same colour represent the two directions of cluster comparison. adj.: adjusted threshold from Table 2.

The cgMLST clustering at described threshold values [12, 13] was set as the reference for the adjustment of clustering thresholds for other methods. Our idea was to define threshold values, which allow for the communication of clustering information between laboratories. Table 2 displays the resulting translation code, which can be applied as follows: in a case in which Laboratory A uses cgMLST analysis with BioNumerics_Moura at the published allele threshold of seven, an allele threshold of eight in cgMLST analysis with Ridom_Ruppitsch used in Laboratory B would result in the best cluster agreement. The corresponding adjusted Wallace coefficients, 97.3 and 98.2 %, can be found in Fig. 5a. If Laboratory C uses SNP analysis with Snippy_EGDe, a threshold of 13 SNPs should be applied to yield comparable clustering to Laboratory A. If Laboratory C, however, uses a different reference genome in SNP analysis, for example a draft MLST CC-specific one (Snippy_draft), a threshold of 12 SNPs will be best suited to fit the clustering from Laboratory A.

**Table 2.** Adjusted thresholds for optimised clustering concordance between cgMLST methods and between cgMLST and SNP methods. Clustering by cgMLST methods at published thresholds [12, 13] (in bold type on the left) was set as reference for the adjustment of clustering thresholds for other methods. The columns show the different comparison methods and the threshold values (alleles or SNPs) at which the greatest possible agreement among the clustering with the respective reference method was achieved based on adjusted Wallace coefficients presented in Fig. 5. As cluster comparison is direction-dependent, the table must be read from the left to the right

			cgMLST			SNP					
						General reference		MLST CC-specific reference			
		**Allele threshold**	Ridom_Ruppitsch	chewBBACA_Ruppitsch	BioNumerics_Moura	Snippy_EGDe	BioNumerics_EGDe	Snippy_closed	Snippy_draft	BioNumerics_closed	BioNumerics_draft
**cgMLST**	Ridom_ Ruppitsch	**7**		6	6	9–10	4	11	12	12	11–12
	chewBBACA_ Ruppitsch		7		7	9–10	4	11	12	12	11–12
	BioNumerics_ Moura		8	7		13	4	12	12	11	11–12
	Ridom_ Ruppitsch	**10**		10	10	15	5	19	19	18	18
	chewBBACA_ Ruppitsch		10		10	15	5	20	18–19	18	18
	BioNumerics_ Moura		11	11		18	6	20	22	19	20

Although use of identical thresholds in different cgMLST methods already led to higher concordance of clustering than use of different cgMLST scheme-specific thresholds, slight adjustment of thresholds (±1 allele) could increase concordance even more (Table 2, Fig. 5). For example, clustering at an allele threshold of seven in BioNumerics_Moura, compared with Ridom_Ruppitsch setting a threshold of eight instead of seven alleles, led to a method concordance of at least 97.3 % (Fig. 5a) instead of only 90.6 % (Fig. 4).

Overall, at an allele threshold of seven, achievable method concordance with cgMLST and SNP methods was at least 90.9 % (Fig. 5a) and at a threshold of ten alleles in cgMLST at least 77.9 % (Fig. 5b).

When using a general reference genome (EGDe) in SNP analysis, threshold values for optimised clustering concordance with cgMLST were lower than with an MLST CC-specific reference. Additionally, thresholds differed between SNP_Snippy_EGDe and SNP_BioNumerics_EGDe, but threshold values were similar for MLST CC-specific approaches irrespective of whether closed or draft references or the two different software tools were applied (Table 2).

#### Practical test of the translation code

**Fig. 6.**
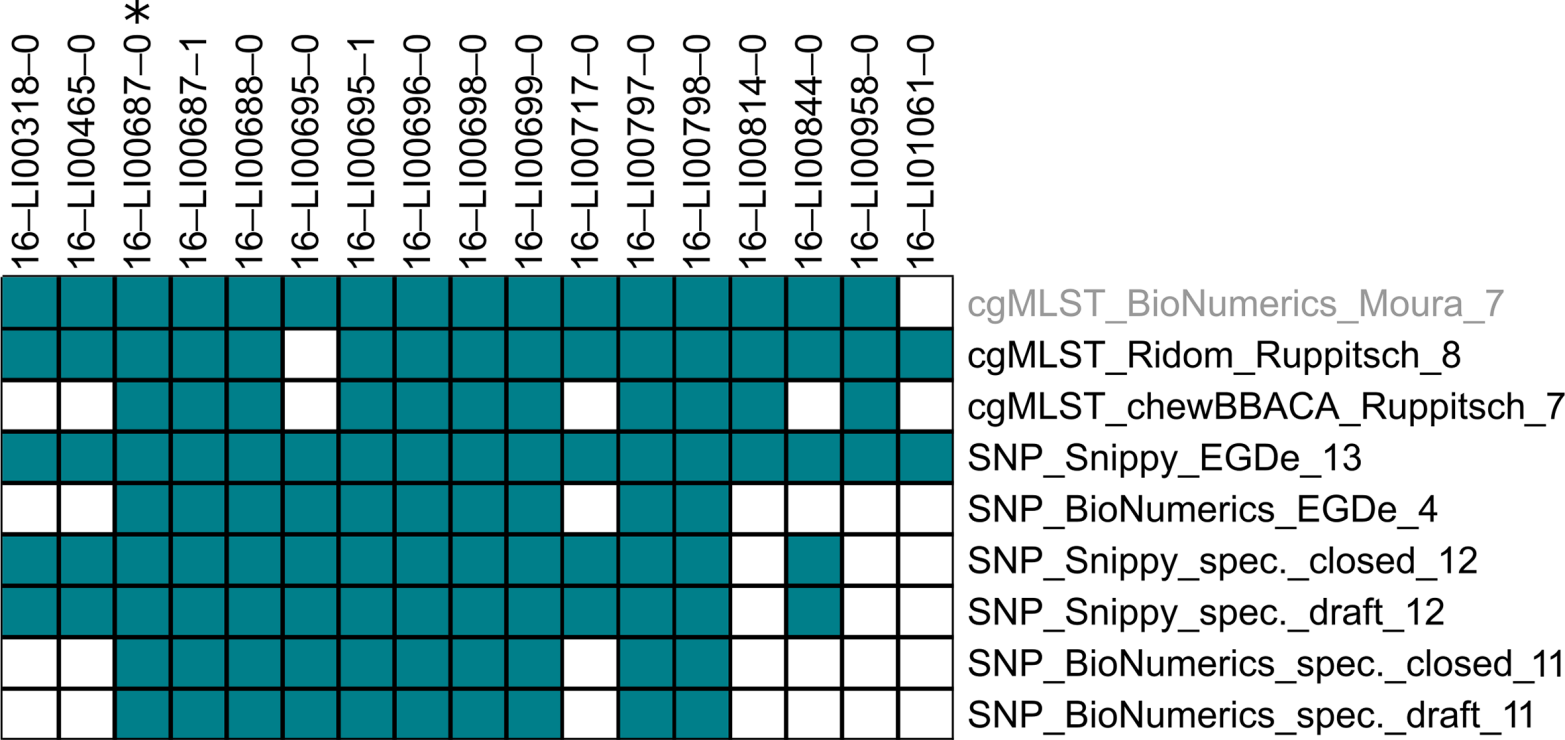
Practical test of the translation code taking a cgMLST cluster of 16 isolates belonging to MLST CC121 as an example. The cgMLST dataset retrieved from BioNumerics_Moura at an allele threshold of seven (grey text colour) was used as reference method for clustering. Labelling on the right, ‘method_threshold’. Upper labels: isolate identifiers. An asterisk indicates the isolate that was used for cluster search in the different methods. Members of a cluster are coloured. Corresponding distance matrices can be found in Supplementary file S3.

For the cgMLST cluster from CC121 retrieved from BioNumerics_Moura at an allele threshold of seven (16 isolates), clustering differed by one to six isolates (median 3.5) when using other methods (Fig. 6).

In contrast, for the cgMLST clusters from CC3 (15 isolates) and CC2 (seven isolates), agreement was perfect, apart from a single isolate that was missing in the clustering results from SNP_Snippy_EGDe. For the clusters from CC9 (26 isolates) and CC8 (eight isolates), exactly the same isolates were found to form a cluster at the adjusted threshold values in all methods.

## Discussion

### cgMLST

Use of the Moura cgMLST scheme mostly resulted in higher allele distances than the Ruppitsch scheme. Given that the number of loci included in the two schemes differs by 47, this was to be expected. Overall correlation of the different cgMLST approaches was high, probably due to the 1261 loci overlap between the two schemes [13]. However, use of the Ruppitsch scheme either in Ridom SeqSphere+or in chewBBACA resulted in slightly different allele distances. This can be attributed to differences in the way the two algorithms work. While the query sequence is compared to the loci via a nucleotide blast (BLASTn) in Ridom SeqSphere+, chewBBACA is centred on the prediction of CDS and a subsequent protein blast (BLASTp). The idea behind making a blastp instead of a blastn is that silent mutations are ignored because they are biologically irrelevant [14]. However, a major problem with blastp arises from frameshifts – either biologically present or due to assembly errors – which can change an entire protein (all amino acids). In a blastn approach, a frameshift is perceived as a single nucleotide change.

Apart from the pure distances between isolates, in outbreak investigations especially, clustering of isolates is important to provide enough evidence for potential epidemiological links. Due to its ease of use and the possibility of a unified nomenclature, gene-by-gene approaches are recommended for that purpose by the PulseNet International global consortium [20, 25]. We, therefore, applied cgMLST together with epidemiologically well-defined clustering threshold values to establish a translation code between different WGS typing approaches (Table 2). It is important to note that the idea of fixed clustering thresholds is controversial, as has been discussed previously [33, 34]. WGS trace-back analysis always has to be used in combination with epidemiological evidence and published thresholds should be seen more as guidelines than as absolute rules [35]. Isolates that fall into a cluster at a certain threshold do not necessarily have to be epidemiologically linked [33]. Nevertheless, threshold values can be a valuable tool for a first delimitation of possibly linked isolates.

Despite the different numbers of loci in the different cgMLST schemes, application of unified thresholds yielded higher clustering concordance than application of scheme-specific thresholds. Slight adjustment of the thresholds could further increase concordance and led to identical clustering in four out of five tests of the translation code. However, method concordance did not reach 100%, even when using the same cgMLST scheme. This shows that not only the agreement on a specific scheme but also on specific software is important to achieve unambiguous comparability of clustering results.

### SNP

While generally applicable typing methods may provide a valuable overview, further analyses on a subgroup of isolates will help to gain deeper insights. A potential outbreak cluster, for instance, can be initially identified by using cgMLST or SNP analysis with a general reference genome. A higher resolution of the closely related isolates within the cluster can be achieved by subsequent SNP analysis with a specific reference genome. To this end, the use of MLST CC-specific references has been proposed [16].

SNP analysis, limited to closely related isolates (in our case isolates of a specific MLST CC) using a closely related reference genome, reduced differences between the tools Snippy and BioNumerics when compared with analysis with a general reference. This indicates that such a restriction to closely related genomes improves method robustness. Additionally, in agreement with results from previous studies [17], using a closed or a draft specific reference genome did not have a decisive effect (neither on distance matrix nor on clustering). Both approaches have advantages and disadvantages. While a closed genome resolves repetitive regions, those will most probably not be present in a draft assembly. This phenomenon could decrease the number of detected SNPs actually present in unresolved regions and close to contig borders in a draft reference genome. Conversely, a draft genome from a certain study population is likely to have a higher degree of similarity to the rest of the isolates than a closed genome from a public repository, which may increase the core genome size and thus potentially the number of detected SNPs. As we have shown above (similar size of core genomes in SNP_Snippy with draft and closed reference genomes), the two effects (closeness and completeness) appeared to offset each other in our dataset. Therefore, if closed reference genomes specific for MLST CCs are not available, draft genomes from the dataset to be analysed can be used equivalently as references without losing analytical accuracy.

As an alternative to reference-based SNP calling, also reference-free, k-mer based approaches exist [36, 37]. They may have the advantage that no bias is introduced due to the selection of a certain reference. However, results are thereby highly dependent on the dataset and more difficult to compare than results derived from standardised, pre-defined references. Therefore, reference-based SNP calling using pre-defined references in the form of MLST CC-specific reference genomes will lead to more standardised results when different datasets are compared.

While there was no difference between different software when using a specific reference genome, differences were large between Snippy and BioNumerics when using a general reference genome. The clustering threshold in the translation code for SNP_BioNumerics, in turn, was generally lower than for SNP_Snippy. As filter settings were similar in the two tools, the reasons for this effect remain unclear. Differences in the size of the core genome used in SNP analysis might have played a role. At this point, a major disadvantage of commercial over open-source tools becomes obvious. Although a closed-source software solution may be easier to use, open-source tools offer higher transparency since they allow for full comprehension of all steps in the analysis and provide intermediate and final results in standardised bioinformatics file formats.

## Conclusions

In case of international disease outbreaks, for instance, one country needs to know whether related strains are found in other countries so that appropriate measures can be taken to prevent human infections. However, different laboratories frequently have different preferences for WGS data analysis. Such missing standards might hamper collaboration between sectors and countries [38]. Although web servers can be used for shared data analysis, the great advantage of local data analysis over submitting results to a web server is that the period between sequencing experiment and analysis results can be influenced. Of course, time also depends on the computational infrastructure at a certain institute. However, especially in time-critical applications like outbreak investigations, this could be a limiting factor. Even when primary analysis is performed locally, use of harmonised methods would open the opportunity to exchange intermediate results, like allele profiles in the case of cgMLST or variant files in the case of SNP analysis. These could then be used for global clustering with little computational effort.

Until harmonisation of methods is achieved, a translation code based on method concordances can work as a first filter to identify typing matches resulting from the different WGS analysis methods. This gives a new perspective for data exchange. The main advantage of our approach is the free choice of analysis tools, provided that there is good concordance with comparison methods. In this way, methods already established in a laboratory can be applied and uptake challenges of a method prescribed by another party are avoided.

Our translation code represents an average over the population structure of *L. monocytogenes* in the food chain in Germany. Despite the predominantly encouraging results from our practical test, we have seen that the WGS analysis methods may show better or worse agreement for individual clusters and different combinations of methods. This is also reflected in the range of adjusted Wallace coefficients achievable (Fig. 5). These coefficients must always be kept in mind when using the translation code, since they provide information about the probability of exact cluster matches between two methods of analysis. If these values are too low, the use of an alternative method on either side should be considered in order to improve adjusted Wallace coefficients before exchanging cluster information. However, good translatability (high clustering concordances at adjusted threshold values) between the majority of tested methods offers the valuable opportunity to minimise the amount of sequence data that needs to be exchanged and individually re-analysed. In this way, processes can be accelerated, which is an enormous advantage, especially in time-critical analyses of supraregional outbreaks.

## Supplementary Data

Supplementary material 1

Supplementary material 2
