# Supplementary material for: Translatability of WGS typing results can simplify data exchange for surveillance and control of Listeria monocytogenes
Source: Microb Genom. 2020 Dec 4;7(1):mgen000491. doi: 10.1099/mgen.0.000491 (PMC8115905; doi:10.1099/mgen.0.000491)
Supplement: Supplementary material 2 [file mgen-7-491-s002.pdf]

Supplement 1: Tools and parameters

Tool	Version	Parameters
Trimmomatic	0.36	ILLUMINACLIP:NexteraPE-PE.fa:2:30:10<br>SLIDINGWINDOW:4:20<br>MINLEN:50
unicycler	0.4.4	--mode conservative<br>--min_fasta_length 300
chewBBACA	2.0.12	--bsr: 0.6 # *BLAST score ratio (BSR) threshold*<br>--ptf Listeria_monocytogenes.trn # *prodigal training file*
GrapeTree	1.4.1	--method distance<br>--missing 0 # *(Number of different alleles)/(Number of loci that present in both genomes)*(Total number of loci)*
Snippy	4.0	mapqual: 60 # *Minimum read mapping quality to consider*<br>basequal: 13 # *Minimum base quality to consider*<br>mincov: 10 # *Minimum site depth to for calling alleles*<br>minfrac: 0 # *Minumum proportion for variant evidence (0=AUTO)*<br>minqual: 100 # *Minumum QUALITY in VCF column 6*<br>maxsoft: 10 # *Maximum soft clipping to allow (default '10')*

Supplement 2: Distance comparison

Visual comparison of the distances derived from different methods.
Each point represents a pairwise difference, with the point's x-value being the distance in method 1 (marked on the x-axis) and the y-value being the distance in method 2 (marked on the y-axis). If the points are found on the diagonal regression line (grey), this indicates a high degree of agreement between the methods (also reflected in a high correlation value).
To highlight how many isolates are clustered differently by different tools, exemplary cluster thresholds for the individual methods are included in the figures, represented by a horizontal or vertical line (red). All points located in the lower left quadrant are assigned to the same cluster by both methods at the thresholds applied. If a point is either above this quadrant or to the right of it, this is a case where the clustering does not match. All points in the upper right quadrant are correctly assigned to no cluster.

1. *cgMLST versus cgMLST*

cgMLST_Ridom_Ruppitsch versus
cgMLST_chewBBACA_Ruppitsch

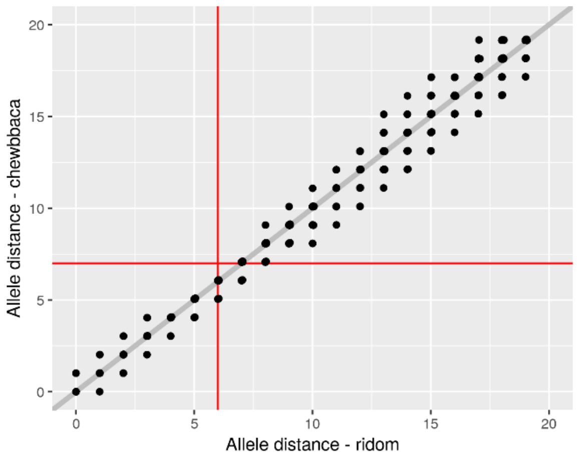

cgMLST_Ridom_Ruppitsch versus
cgMLST_BioNumerics_Moura

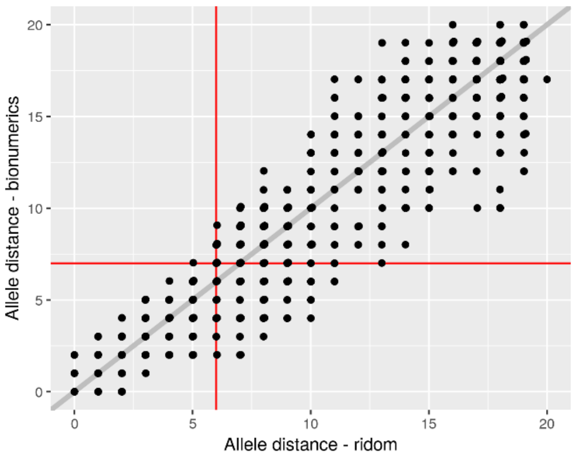

cgMLST_chewBBACA_Ruppitsch versus
cgMLST_BioNumerics_Moura

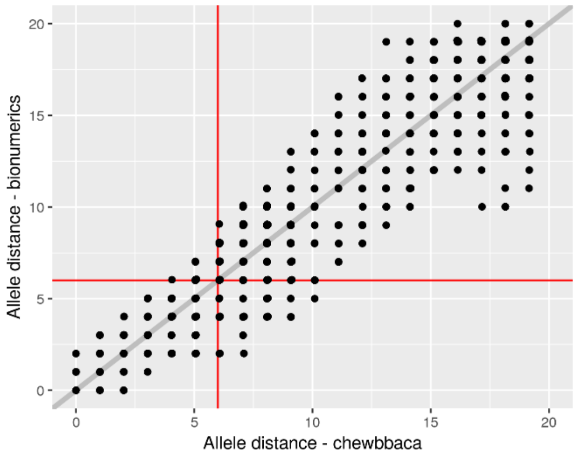

2. SNP versus SNP

2.1. Software-wise

SNP_Snippy_spec._closed versus
SNP_BioNumerics_spec._closed

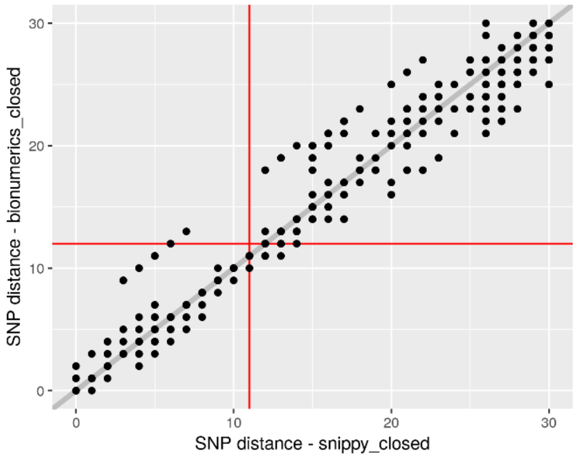

SNP_Snippy_spec._draft versus
SNP_BioNumerics_spec._draft

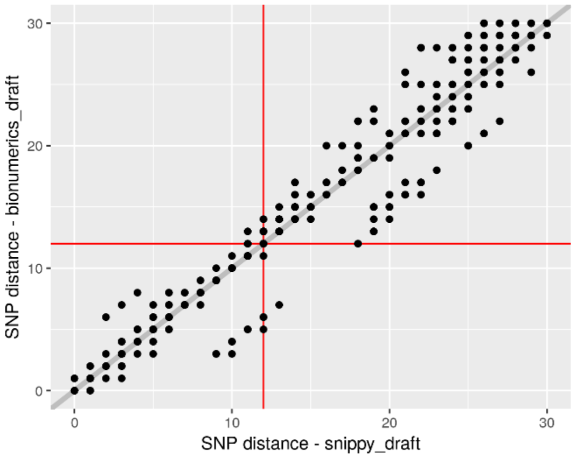

SNP_Snippy_EGDe versus
SNP_BioNumerics_EGDe

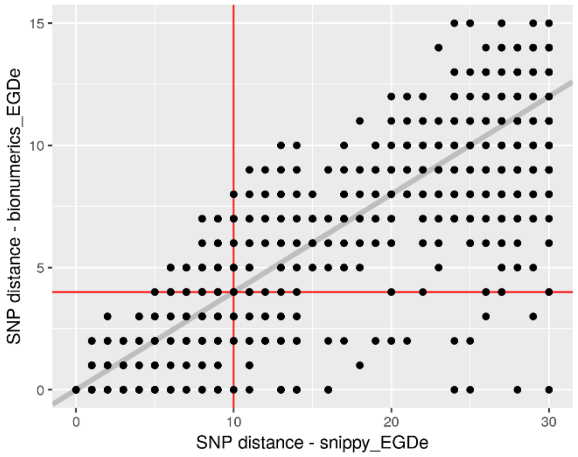

2.2. Reference genome-wise

SNP_Snippy_spec._closed versus
SNP_Snippy_spec._draft

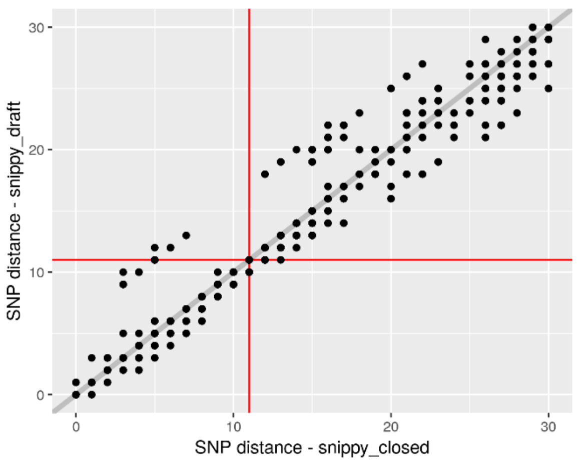

SNP_Snippy_spec._closed versus
SNP_Snippy_EGDe

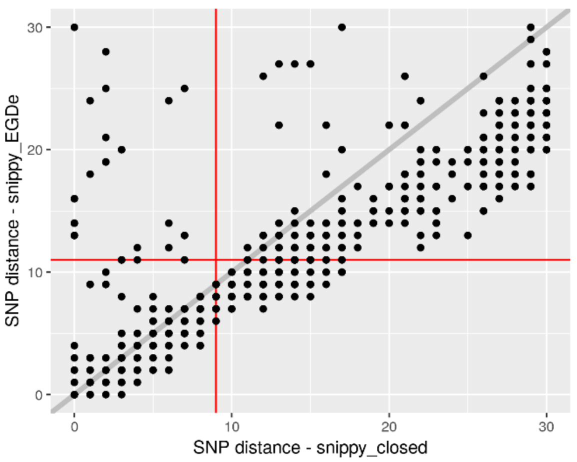

SNP_BioNumerics_spec._closed versus
SNP_BioNumerics_spec._draft

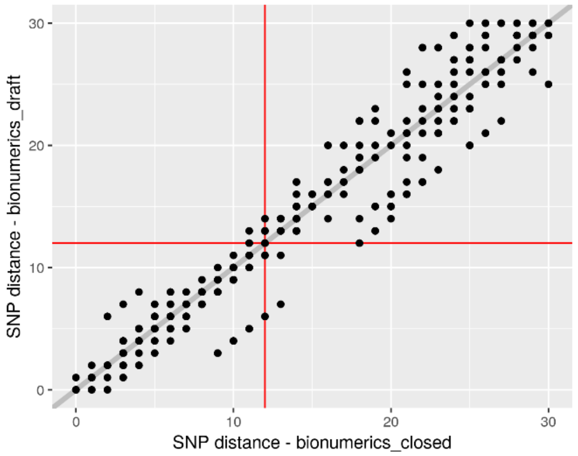

SNP_BioNumerics_spec._closed versus
SNP_BioNumerics_EGDe

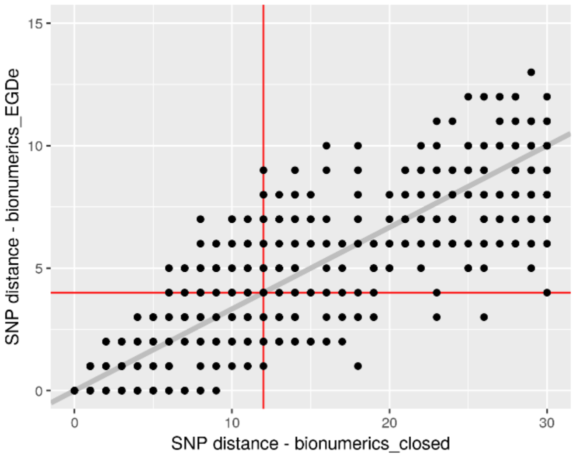

Supplement 3: Distance matrices for the translation code test on MLST CC121 isolates

Allele distances and SNP distances were compared for all applied methods.
Distances are coloured according to the method-specific (translational) threshold. White: distance value within threshold; red: above threshold; orange: borderline case where actual cgMLST distances are above thresholds (e.g. 7.2) but distances were rounded for better visibility.

The reference method was cgMLST_BioNumerics_Moura with an allele threshold of seven. Correct and wrong clustering of the different test methods is indicated through the colour code of isolate identifiers. Blue: correctly identified as a member of the cluster (compared to the reference method); black: incorrectly not assigned to cluster; red: correctly not assigned to cluster; pink: incorrectly assigned to cluster.

cgMLST_Moura_7 (reference method)

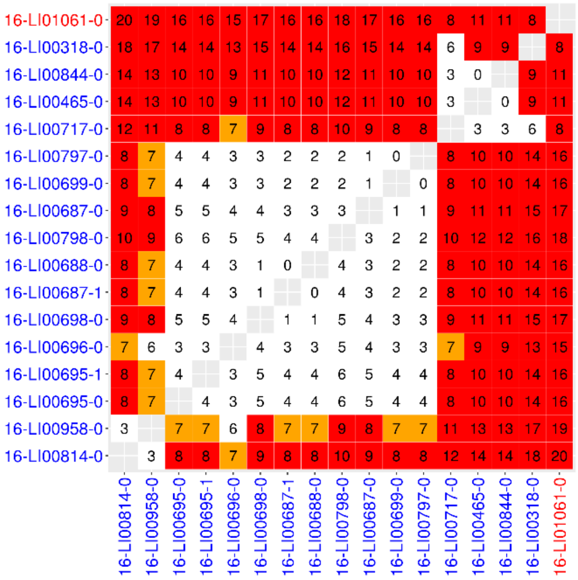

cgMLST_Ridom_Ruppitsch_8

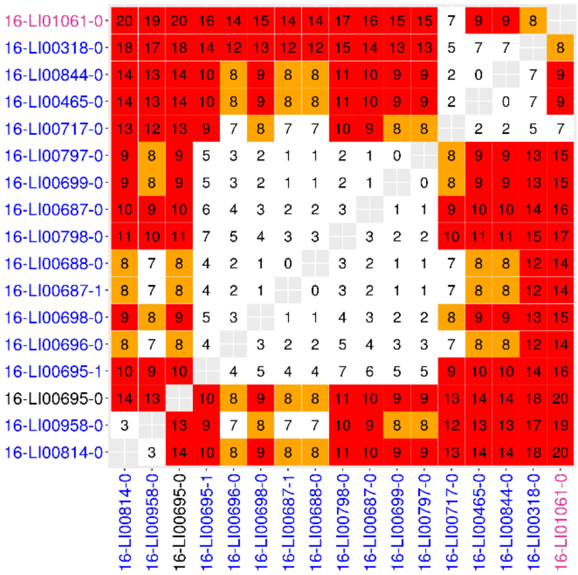

cgMLST_chewBBACA_Ruppitsch_7

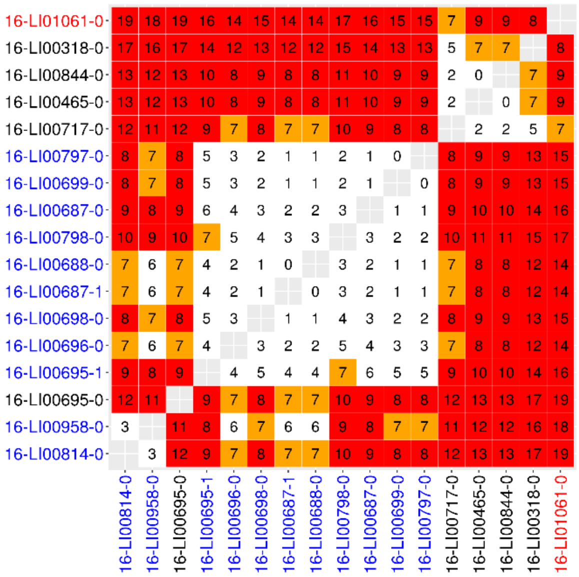

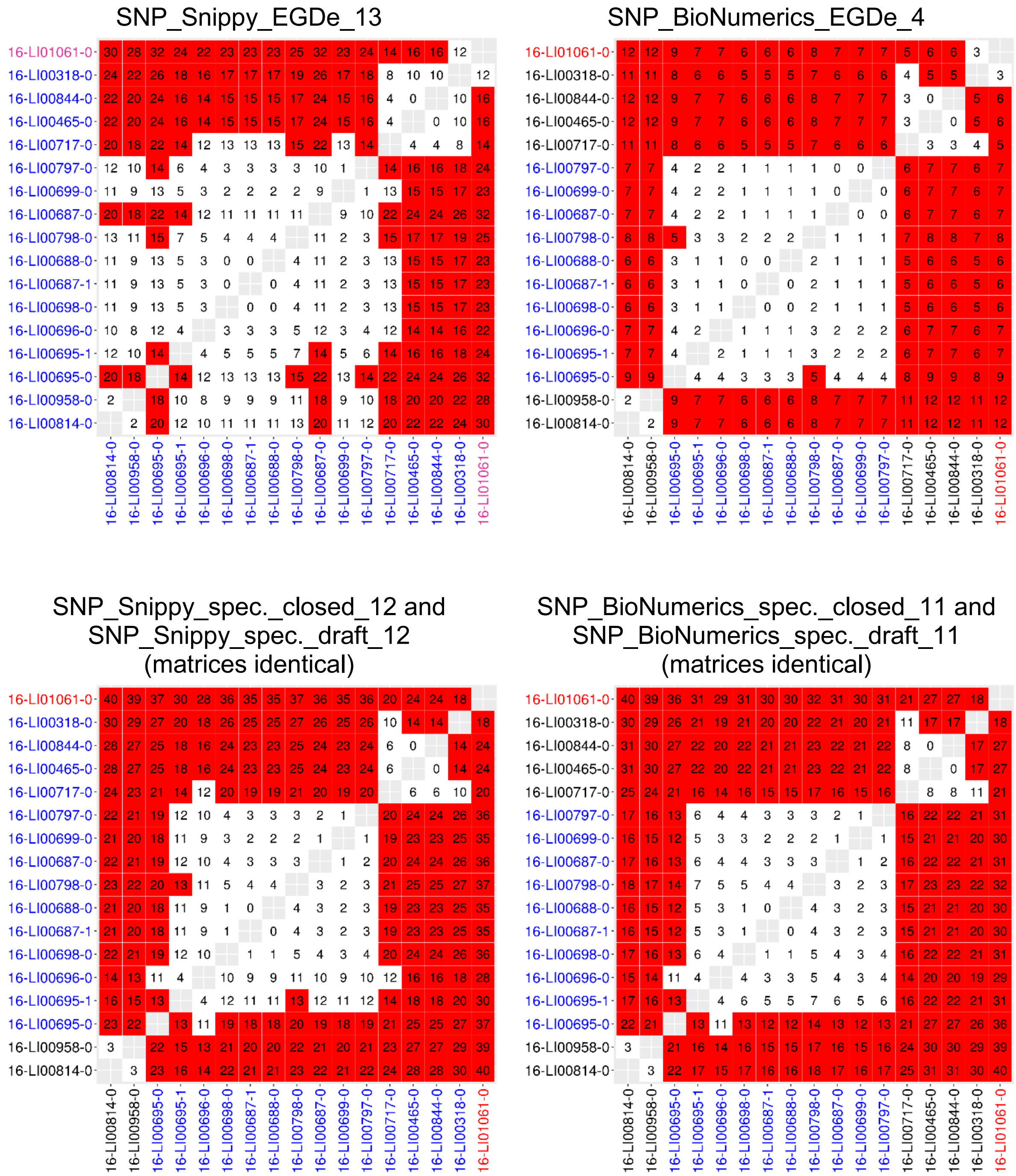
SNP_Snippy_EGDe_13
SNP_BioNumerics_EGDe_4
SNP_Snippy_spec._closed_12 and SNP_Snippy_spec._draft_12 (matrices identical)
SNP_BioNumerics_spec._closed_11 and SNP_BioNumerics_spec._draft_11 (matrices identical)
16-LI01061-0
16-LI00318-0
16-LI00844-0
16-LI00465-0
16-LI00717-0
16-LI00797-0
16-LI00699-0
16-LI00687-0
16-LI00798-0
16-LI00688-0
16-LI00687-1
16-LI00698-0
16-LI00696-0
16-LI00695-1
16-LI00695-0
16-LI00958-0
16-LI00814-0
